# Supplementary material for: New Caledonian Crows Use Mental Representations to Solve Metatool Problems
Source: Curr Biol. 2019 Feb 18;29(4):686–692.e3. doi: 10.1016/j.cub.2019.01.008 (PMC6384166; doi:10.1016/j.cub.2019.01.008)
Supplement: Document S1. Figure S1 and Tables S1–S4 [file mmc1.pdf]

Current Biology, Volume *29*

# Supplemental Information

# New Caledonian Crows Use Mental Representations to Solve Metatool Problems

**Romana Gruber, Martina Schiestl, Markus Boeckle, Anna Frohnwieser, Rachael Miller, Russell D. Gray, Nicola S. Clayton, and Alex H. Taylor**

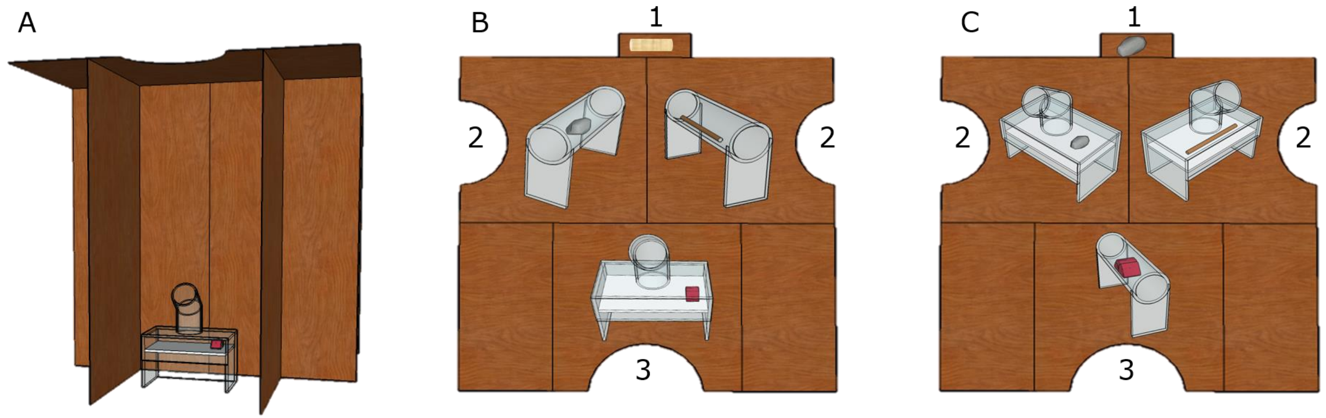


**Figure S1. Diagram of the wooden box used to visually shield off the apparatuses from each other (related to Figure 1).** Viewed from the side (A), and from a bird's eye view (B & C). 1) Initial tool starting position, 2) Sub-goals 3) Goal. Red block indicates meat position.

| | | Number of Blocks for Training Stage 1 | | | | | |
|---|---|---|---|---|---|---|---|
| **Year** | **Individuals** | **1** | **2** | **3** | **4** | **5** | **6** |
| 2017 | **Janis** | 9/12 | 9/12 | | | | |
| 2017 | **David** | 8/12 | 10/12 | | | | |
| 2017 | **Freddie** | 11/12 | 10/12 | | | | |
| 2017 | **Elvis** | 10/12 | 8/12 | | | | |
| 2017 | **Bob** | 10/12 | 11/12 | | | | |
| 2017 | **Aretha** | 7/12 | 6/12 | 6/12 | 5/12 | 8/12 | 10/12 |
| 2018 | **Mercury** | 6/10 | 8/10 | 6/10 | 8/10 | 9/10 | |
| 2018 | **Io** | 7/10 | 10/10 | | | | |
| 2018 | **Neptune** | 7/10 | 10/10 | | | | |
| 2018 | **Triton** | 8/10 | 6/10 | 9/10 | 6/10 | 7/10 | 10/10 |
| 2018 | **Mars** | 7/10 | 9/10 | | | | |
| 2018 | **Saturn** | 10/10 | 9/10 | | | | |
| 2018 | **Uranus** | 7/10 | 8/10 | 8/10 | | | |
| 2018 | **Venus** | 7/10 | 6/10 | 8/10 | 7/10 | 9/10 | |

**Table S1. Blocks required for 2017 and 2018 birds for Training Stage 1 (related to Figure 2).** Note birds were given blocks of 12 trials in 2017, and needed to reach a criterion of 18/24, while in 2018 they were given blocks of 10 trials, and needed to reach a criterion of 16/20. The colours indicate a failed block (red) and a successfully solved block (green).

| | | Number of Blocks for Training Stage 2 | | | | | | | | | | | | | | | | | |
|---|---|---|---|---|---|---|---|---|---|---|---|---|---|---|---|---|---|---|---|
| **Year** | **Individuals** | **1** | **2** | **3** | **4** | **5** | **6** | **7** | **8** | **9** | **10** | **11** | **12** | **13** | **14** | **15** | **16** | **17** | **18** |
| 2017 | **Janis** | 5/12 | 9/12 | 7/12 | 11/12 | | | | | | | | | | | | | | |
| 2017 | **David*** | 7/12 | 10/12 | | | | | | | | | | | | | | | | |
| 2017 | **Freddie** | 7/12 | 9/12 | 5/12 | 5/12 | 7/12 | 10/12 | 8/12 | | | | | | | | | | | |
| 2017 | **Elvis** | 7/12 | 9/12 | 5/12 | 7/12 | 10/12 | 7/12 | 9/12 | 8/12 | 10/12 | | | | | | | | | |
| 2017 | **Bob** | 6/12 | 3/12 | 5/12 | 5/12 | 7/12 | 6/12 | 6/12 | 6/12 | 7/12 | 8/12 | 10/12 | | | | | | | |
| 2017 | **Aretha** | 6/12 | 5/12 | 5/12 | 8/12 | 4/12 | 6/12 | 11/12 | 9/12 | | | | | | | | | | |
| 2018 | **Mercury** | 6/10 | 7/10 | 5/10 | 4/10 | 7/10 | 7/10 | 6/10 | 6/10 | 8/10 | 8/10 | 7/10 | 8/10 | 10/10 | 8/10 | 9/10 | | | |
| 2018 | **Io** | 10/10 | 7/10 | 6/10 | 7/10 | 8/10 | 7/10 | 7/10 | 5/10 | 10/10 | 7/10 | | | | | | | | |
| 2018 | **Neptune** | 8/10 | 8/10 | 4/10 | 6/10 | 8/10 | 9/10 | | | | | | | | | | | | |
| 2018 | **Triton** | 6/10 | 8/10 | 5/10 | 1/10 | 4/10 | 8/10 | 7/10 | 4/10 | 5/10 | 5/10 | 9/10 | 7/10 | 7/10 | 9/10 | 10/10 | | | |
| 2018 | **Mars** | 9/10 | 10/10 | 9/10 | 8/10 | | | | | | | | | | | | | | |
| 2018 | **Saturn** | 6/10 | 8/10 | 10/10 | 10/10 | 9/10 | | | | | | | | | | | | | |
| 2018 | **Uranus** | 7/10 | 7/10 | 10/10 | 3/10 | 6/10 | 10/10 | | | | | | | | | | | | |
| 2018 | **Venus** | 5/10 | 5/10 | 6/10 | 4/10 | 6/10 | 7/10 | 5/10 | 7/10 | 8/10 | 5/10 | 7/10 | 7/10 | 8/10 | 6/10 | 8/10 | 6/10 | 8/10 | 9/10 |

*David reached criterion in 17 out of 24 correct trials due to time constraints.

**Table S2. Blocks required for 2017 and 2018 birds for Training Stage 2 (related to Figure 2).** Note birds were given blocks of 12 trials in 2017, and needed to reach a criterion of 18/24, while in 2018 they were given blocks of 10 trials, and needed to reach a criterion of 16/20. The colours indicate a failed block (red) and a successfully solved block (green).

| | Training | Experiment 1: Sub-goal Representation | | | Experiment 2: Goal Representation | Experiment 3: Apparatus Representation | | |
|---|---|---|---|---|---|---|---|---|
| **Choices** | | **Stick** | **Stone** | **Combined** | | **Stick** | **Stone** | **Combined** |
| **Correct** | 69% | 66.26% | 48.08% | 55.7% | 61.55% | 85.32% | 59.83% | 72.58% |
| **1st action error** | NA | 27.91% | 46.68% | 38.82% | 30.18% | 13.17% | 37.38% | 25.26% |
| **2nd action error** | NA | 1.94% | 1.75% | 1.83% | 0% | 1.59% | 0.56% | 1.07% |
| **3rd action error** | NA | 3.88% | 3.50% | 3.66% | 8.27% | 0% | 2.23% | 1.12% |
| **Adults–correct** | 73.81% | 69.85% | 40.42% | 51.84% | 58.84% | 90% | 56.67% | 65% |
| **Juveniles-correct** | 67.52% | 69.27% | 58.14% | 61.39% | 66.39% | 82.50% | 61.02% | 66.45% |

**Table S3. Percentages of correct choices and errors within Training and the three experiments (related to Figures 2, 3 and 4).**

| Individuals | Stick Condition | Stone Condition |
| --- | --- | --- |
| Aretha | 30% | 37.8% |
| Freddie | 14.3% | 58.1% |
| Elvis | 48.8% | 35% |
| Janis | 40.9% | 35% |
| David | 50% | 2.5% |
| Bob | 50% | 57.5% |

**Table S4. Inspection frequency of the apparatuses with tool in beak by the 2017 crows, for both conditions (related to Figure 2).**
