# Supplementary material for: New Caledonian Crows Use Mental Representations to Solve Metatool Problems
Source: Curr Biol. 2019 Feb 18;29(4):686–692.e3. doi: 10.1016/j.cub.2019.01.008 (PMC6384166; doi:10.1016/j.cub.2019.01.008)
Supplement: Document S2. Article plus Supplemental Information [file mmc4.pdf]

Report

Current Biology

New Caledonian Crows Use Mental Representations to Solve Metatool Problems

Highlights

- Crows solved metatool problems where each stage was out of sight of the others
- Crows avoided distractor apparatuses during problem-solving
- This shows crows mentally represent the goals and sub-goals of metatool problems
- Crows can preplan three behaviors into the future while using tools

Authors

Romana Gruber, Martina Schiestl, Markus Boeckle, ..., Russell D. Gray, Nicola S. Clayton, Alex H. Taylor

Correspondence

rgru908@aucklanduni.ac.nz

In Brief

Gruber et al. presented metatool problems to New Caledonian crows, where each stage of the problem was out of sight of the others. Crows had to mentally represent the location and identities of tools and apparatuses to solve the problems correctly. These results show that New Caledonian crows can preplan several behaviors ahead while using tools.

Gruber et al., 2019, Current Biology *29*, 686–692
February 18, 2019 © 2019 The Authors. Published by Elsevier Ltd.
https://doi.org/10.1016/j.cub.2019.01.008

Current Biology
Report

New Caledonian Crows Use Mental Representations to Solve Metatool Problems

Romana Gruber,[1,6,*] Martina Schiestl,[1,2,5] Markus Boeckle,[3,4,5] Anna Frohnwieser,[3] Rachael Miller,[3] Russell D. Gray,[1,2] Nicola S. Clayton,[3] and Alex H. Taylor[1]
[1]School of Psychology, University of Auckland, 23 Symonds Street, Auckland 1010, New Zealand
[2]Max Planck Institute for the Science of Human History, Kahlaische Strasse 2, 07745 Jena, Germany
[3]Department of Psychology, University of Cambridge, Downing Street, Cambridge CB2 3EB, UK
[4]Department of Psychotherapy, Bertha von Suttner University, Matthias-Corvinus-Straße 15, 3100 St. Pölten, Austria
[5]These authors contributed equally
[6]Lead Contact
*Correspondence: rgru908@aucklanduni.ac.nz
https://doi.org/10.1016/j.cub.2019.01.008

SUMMARY

One of the mysteries of animal problem-solving is the extent to which animals mentally represent problems in their minds. Humans can imagine both the solution to a problem and the stages along the way [1–3], such as when we plan one or two moves ahead in chess. The extent to which other animals can do the same is far less clear [4–25]. Here, we presented New Caledonian crows with a series of metatool problems where each stage was out of sight of the others and the crows had to avoid either a distractor apparatus containing a non-functional tool or a non-functional apparatus containing a functional tool. Crows were able to mentally represent the sub-goals and goals of metatool problems: crows kept in mind the location and identities of out-of-sight tools and apparatuses while planning and performing a sequence of tool behaviors. This provides the first conclusive evidence that birds can plan several moves ahead while using tools.

RESULTS AND DISCUSSION

From Köhler's early work on insight in chimpanzees [25] through to contemporary animal problem-solving studies investigating water displacement [26–28], sequential problem-solving [7, 8, 19, 23, 29, 30], hook-making [23, 31], connectivity [19, 32], gravity [32–35], and planning [18, 22, 36, 37], one key question has been the extent to which animals use mental trial and error. Dennett [38] famously referred to animals with this ability as Popperian creatures because their "hypotheses die in their stead." That is, by being able to mentally represent different states of the world and the potential outcome of actions directed toward changing these states, an animal can try out different courses of action in their heads and then avoid ones that might kill them, or reduce their chances of reproduction, in the real world. While such imaginings have a clear adaptive value, many famous examples of animal problem-solving can be explained by mechanisms other than mental trial and error, such as perceptual-motor feedback loops [7, 39, 40], chaining [41, 42], resurgence [43], and trial-and-error learning [44]. At present, therefore, we have a limited understanding of the planning capabilities of animals in general [4–6, 9–17, 45], let alone their capacity to plan several moves ahead while using tools [4, 7, 8, 18, 19, 21, 23].

New Caledonian (NC) crows are a perfect species to test this possibility, given their complex tool behaviors in the wild [46] and their ability to solve problems involving long sequences of tool-related behavior [7, 8]. While research to date has shown that NC crows can solve metatool problems [7, 8, 47], it is not clear whether these crows are mentally representing and planning out behaviors or are solving the problem on a more moment-by-moment basis using chaining and perceptual-motor feedback loops [7, 40, 48, 49]. Here, we tested between these hypotheses by presenting a series of metatool problems to these crows where each stage was out of sight of the other. Additionally, we added a distractor apparatus containing a tool. In experiment 1, crows had to plan using mental representations of the sub-goals of the problem (the location and identity of a functional tool and a distractor tool) (Figure 1A). In the stick condition, crows had to use a stick to pull a stone from a tube (functional sub-goal), while ignoring a tube containing another stick (distractor sub-goal), and then use this stone to release food from a platform apparatus (goal). The stone condition was the mirror of this, with the crows using a stone to get a stick, which could be used to get food from a tube. In experiment 2, crows had to plan using mental representations of both the sub-goals and the goal of the problem (sub-goals, location and identity of a functional tool and a distractor tool; goal, the identity of the goal apparatus) (Figure 1B). Here, crows were given three different trial types in any one block of trials: the stick and stone conditions and a novel "shortcut" condition, where crows had to take a stone directly to a food-baited platform (goal) while ignoring the sub-goals (sticks placed in the tube and platform apparatus, which could not be used to gain food from the platform). This experiment was run to confirm that crows were representing the final goal of the problem as well as the sub-goals. If crows were not representing the final goal, we expected them to use the available tool to try to gain access to one of the tools at the sub-goal stage rather than take the tool directly to the final goal. In experiment 3, crows had to switch from planning using mental representations of the position and location of the *tools* to planning using the position and

 © 2019 The Authors. Published by Elsevier Ltd.
This is an open access article under the CC BY license (http://creativecommons.org/licenses/by/4.0/).

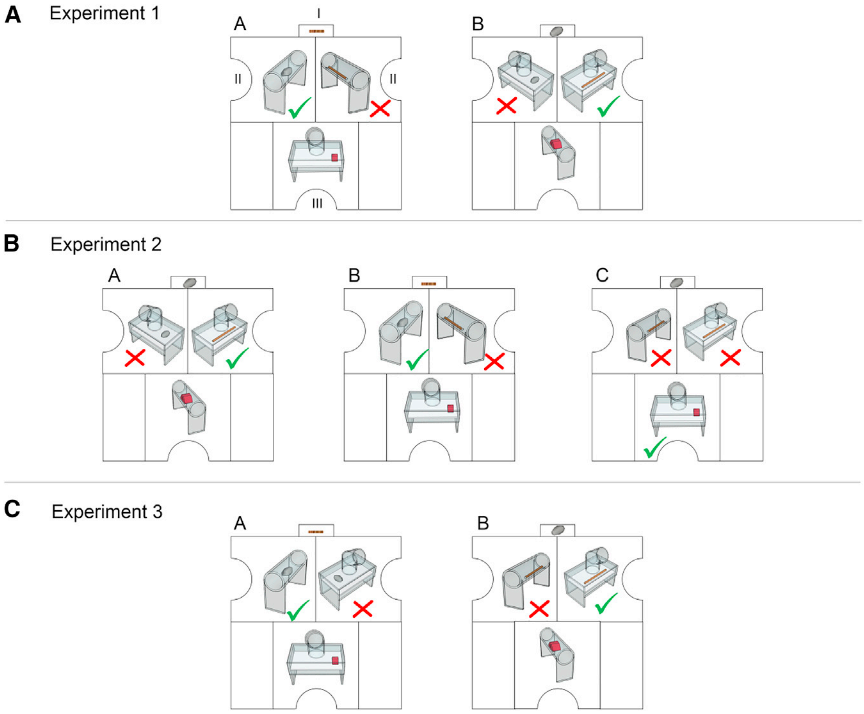

Figure 1. Experiments 1–3 with Each Condition

(i) Initial tool positioned in the small compartment in front of the shield. (ii) Apparatuses containing the tools in the left and right compartments (green tick and red cross indicate the correct tool and the distractor apparatus). (iii) Apparatus baited with meat (red block).

(A) Experiment 1: Stick condition (A): Subjects must use the short stick to get the stone (left), which can then be dropped into the collapsing trap-platform to get the meat, while avoiding the distractor (the long stick, right). Stone condition (B): Subjects must use the stone to get the long stick (right), which can then be used to get the meat from the long tube, while avoiding the distractor (the stone, left).

(B) Experiment 2: Stone condition (A): Same as in Experiment 1. Stick condition (B): Same as in Experiment 1. Shortcut (C): Subjects must take the stone immediately to the final apparatus baited with meat while ignoring both distractor apparatuses.

(C) Experiment 3: Stick condition (A): To get the meat from the platform, subjects must take the stick to the tube and extract the stone while ignoring the platform apparatus. Stone condition (B): Subjects must take the stone to the platform apparatus while ignoring the stick tool in the stick apparatus. Using the stone on the platform apparatus released the long stick, which could be used to get meat from the tube. The black lines indicate the outline of the wooden shield.

See also Figure S1 and Data S1.

location of the *apparatuses* (Figure 1C). Crows were given a stick condition, where they needed to take a stick to get a stone from a tube (functional sub-goal), while ignoring a stone in a platform apparatus (distractor sub-goal), and then use the stone to release food from a platform apparatus (goal). The stone condition was a mirror of this: crows had to use a stone to get a stick from a platform apparatus to get food from a tube, while ignoring a stick in a tube apparatus. This tested whether the crows could spontaneously plan while representing information they had not had experience encoding (the identity *and location* of the apparatus) (Video S1).

If crows had solved past metatool problems without planning, we expected them to fail across our three experiments and either not innovate the new metatool behaviors, not complete the behavioral sequence, or not perform above chance when deciding whether to take the tool to the functional sub-goal, distractor sub-goal, or final goal. In contrast, if NC crows are capable of using mental representations to plan a series of tool behaviors, in each experiment we expected them to be able to complete the sequence and perform metatool tool use to get the correct tool, while ignoring the distractor tool or apparatus.

On experiment 1, 14 crows were tested. Six were tested in 2017, with three being presented with the stone metatool problem first and three the stick metatool problem first. In 2018, we presented a further eight crows with the stick problem then the stone problem. Of the 11 crows that were first presented with the stick metatool problem, 4 reached a criterion of 16/20 with few errors (2 birds from 2017, 2 from 2018, range 0–5; see Figure 2A). A further three crows reached criterion within 40 trials, and one reached criterion in 43 trials (one bird from 2017, three from 2018). All 11 crows were then given the stone problem as their second condition. Two crows reached the criterion of 16/20 trials within 40 trials (one from 2017, one from 2018), while two others did so in their 43rd trial and 46th trial, respectively (one from 2017, one from 2018) (Figure 2B). None of the three 2017 crows that received the stone problem first reached criterion within 40 trials (Figure 2B). However, when these crows were then given the stick condition second, they reached criterion in 22, 23, and 24 trials respectively (Figure 2A).

Experiment 2 was presented to the eight crows in our 2018 field season. Here, crows were given one of three trial types within each block of trials: a stick metatool trial, a stone metatool trial (both the same as in experiment 1), or a shortcut trial. In shortcut trials, crows had to take a stone to get food directly from the platform apparatus (goal) while ignoring two sticks placed inside the two sub-goal apparatuses (tube and platform), as these sticks were non-functional for the overall goal. If crows were not representing the final goal, we predicted they would attempt to use the available tool to gain one of the tools at the sub-goal stage instead of taking the tool directly to the final goal. Thus, to pass this experiment, crows had to plan using mental representations of both the goal and sub-goals of the problem across the three trial types presented to them. Crows were given blocks with these three trial types presented in a pseudorandomized order. Three of the eight birds solved the problem within 60 trials (20 of each condition): Neptune did so in 25 trials, Mars in 26 trials, and Triton in 39 trials. One other bird, Mercury, reached criterion in 69 trials (Figure 3). Clearly, this was a more difficult problem than experiment 1 as crows had to encode more information, namely not only the location

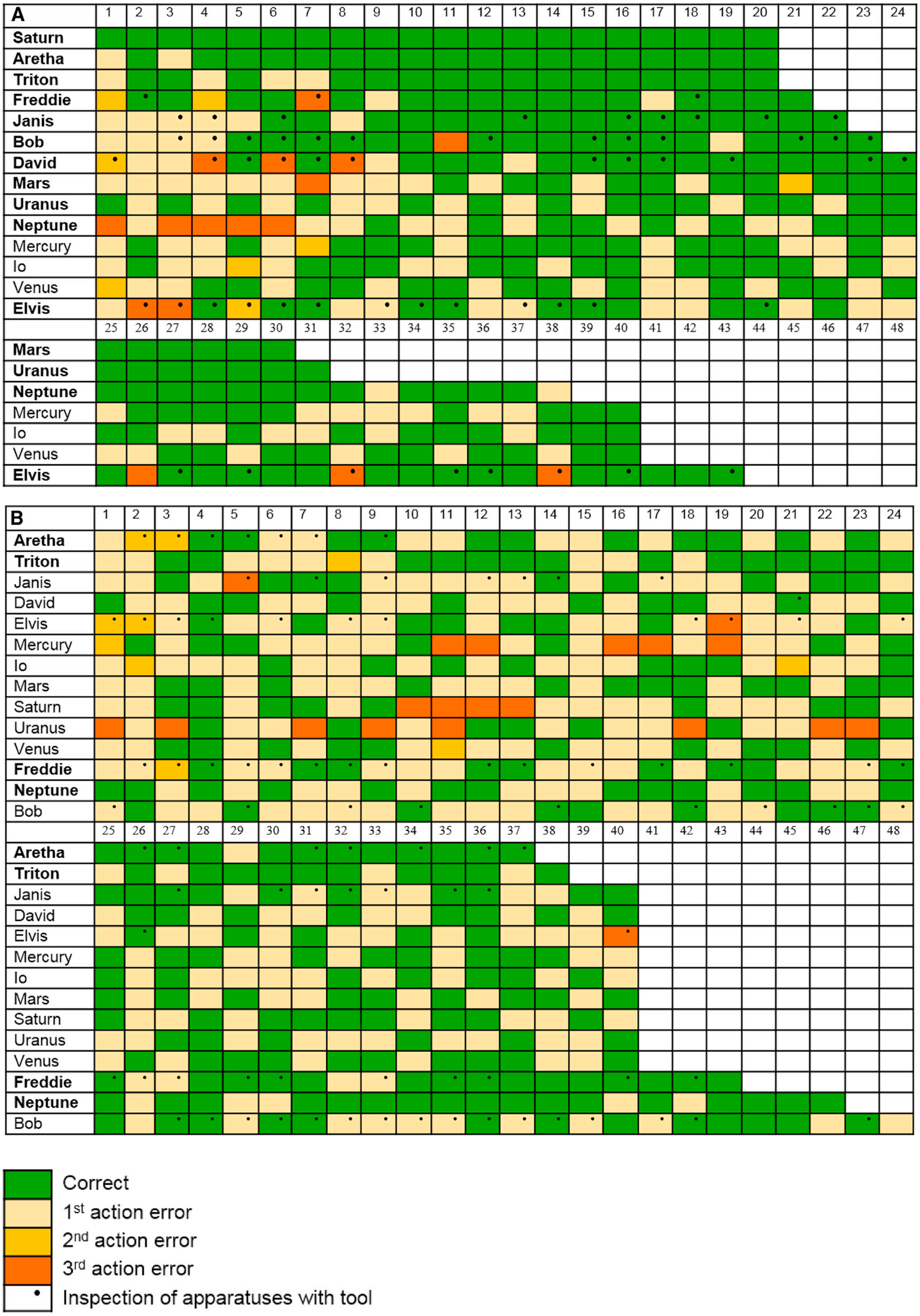

Figure 2. Performance of Crows on Experiment 1

(A and B) Stick condition (A) and stone condition (B). Crows that solved the task are marked in bold.
See also Tables S1–S4 and Data S1.

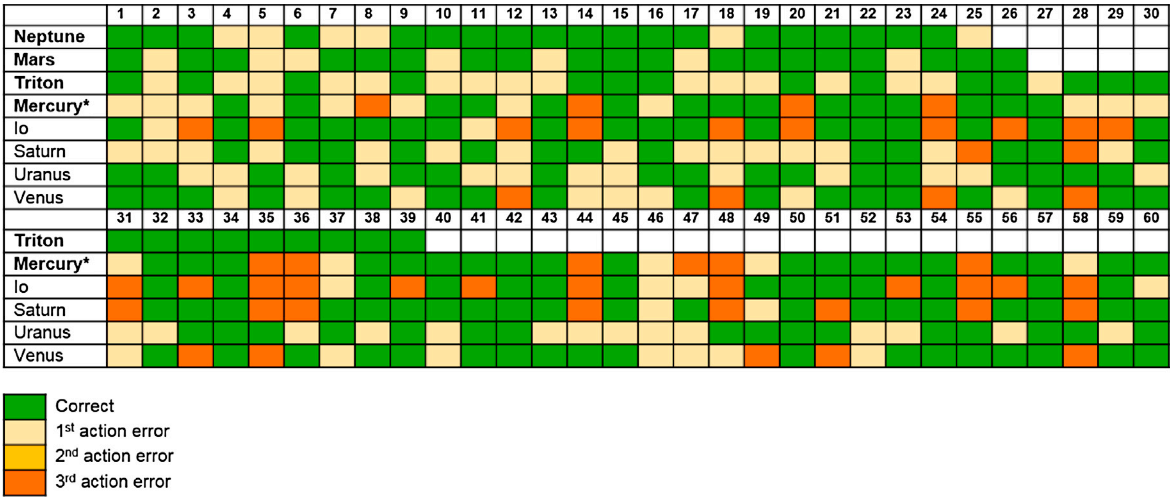

Figure 3. Performance of Crows on Experiment 2 in the First 60 Trials
Crows that solved the task are marked in bold. Mercury solved experiment 2 in trial 69 (*).
See also Table S3 and Data S1.

and identity of the two tools but also the identity of the goal apparatus as well.

Experiment 3 was presented to three of four crows that passed experiment 2 (one crow was excluded due to procedural problems). In all experiments to this point, crows had to plan using mental representations of the locations and identity of the two out-of-sight tools. In this experiment, this changed: for each sub-goal, the same tool was placed in either a functional or non-functional apparatus. Thus, in the stick condition, crows had to take a stick to gain a stone from a tube (functional sub-goal), while ignoring a stone in a platform (distractor sub-goal), and then take the stone to extract food from a platform (goal). In the stone condition, crows had to use a stone to gain a stick from a platform (functional sub-goal), while ignoring a stick in a tube (distractor sub-goal), and then use this stick to gain food. Crows, therefore, had to plan using representations of both the identity and location of the apparatuses, which they had never had to do before. Crows were given 20 trials of each condition. The three crows reached criterion within the first 20 trials of the stick condition, scoring 18, 17, and 16 out of 20 (Figure 4A). In contrast, no crow reached criterion within 20 trials in the stone condition, and only one reached criterion within 60 trials (Figure 4B).

A key signature of human foresight is preplanning, where a mental plan is formed before movements begin to be executed. This differs from online planning, where planning occurs during task-related movements [1, 3]. Crows could have used online planning in our 2017 experiment by picking up the tool and then inspecting one apparatus, without actually interacting with it, before moving onto another. We re-analyzed the performance of the 2017 crows that reached criterion at either task of experiment 1 after excluding trials where crows inspected one apparatus while holding the tool before switching to the other. Instead, we examined only those trials where crows took the tool and immediately interacted with one apparatus. This allowed us to examine how well the 2017 crows performed in trials where only preplanning, rather than online planning, could have been used to solve the problem. In the stick condition, two crows were still significant when we excluded trials where crows inspected the problem while holding a tool. Aretha scored 12/14 correct trials (Binomial choice, $p = 0.013$), and Freddie scored 14/18 correct trials (Binomial choice, $p = 0.031$). Thus, when looking at 2017 trials where online planning did not occur, clear evidence of preplanning emerged. This 2017 finding is mirrored in our 2018 data, where two crows solved the problem with few errors (Saturn, 20/20; Triton, 16/20). In 2018, during our training stages (see Method Details), we gave the crows experience that a trial would be stopped if they picked up a tool and then inspected an apparatus without interacting with it. If crows then attempted to inspect while holding a tool during experiments 1–3, this was counted as an incorrect choice. This highly stringent criterion meant that crows were unable to use online planning across experiments 1–3 in 2018.

Our results provide conclusive evidence that some NC crows can preplan using mental representations of the sub-goals and goals of a metatool problem (experiments 1 and 2) and then spontaneously switch from preplanning using mental representations of different tools to representations of different apparatuses (experiment 3). In experiment 1, we found that most of the crows we tested solved a stick problem, where they had to use a stick tool to get a stone tool and then use the stone to get food while avoiding a distractor object (another stick). This was despite crows not being able to view more than one stage of the problem at a time. Four of the crows we tested showed clear evidence of preplanning. Experiment 2 showed that NC crows can also represent both the goal and sub-goal of a metatool problem while preplanning. Four out of eight crows were able to track both of these features of the problem, with one crow solving this task in 25 trials and one in 26 trials. However, this was clearly a much harder problem for the crows, possibly because they had to mentally represent more information. This may explain why the crows struggled despite having had

CellPress

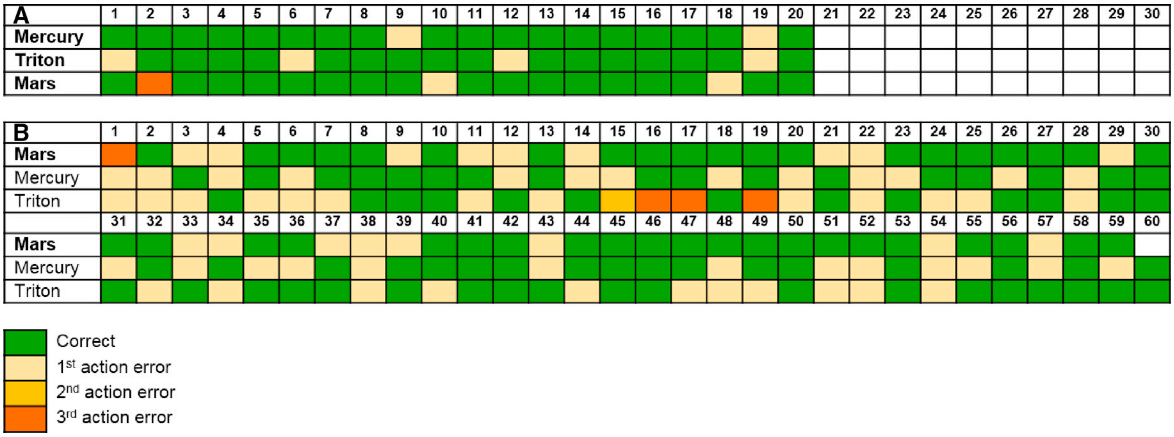

Figure 4. Performance of Crows on Experiment 3
(A and B) Stick condition (A) and stone condition (B). Crows that solved the task are marked in bold.
See also Table S3.

experience of two of the three problems presented already and why the individual that had done best at experiment 1, Saturn, actually failed this experiment. Finally, experiment 3 shows that NC crows can spontaneously preplan using novel information. Crows had not been required to mentally represent two apparatus types and then choose between them at any point in our experiment. However, when presented with a stick problem where they had to take a stick to gain a stone from a tube while ignoring a stick in a platform, the three crows we tested immediately preplanned using a representation of the apparatus type and location rather than the tool type. To our knowledge, these results are the first conclusive evidence that birds can plan several steps ahead while using tools. Our study clearly rules out the alternative explanations suggested for past bird performances on sequential tool problems, where all the components of the problem have been visible [7, 8, 29, 47, 48, 50, 51].

The crows clearly needed considerable experience of mentally representing tools to produce the metatool performances we observed. Though most crows quickly solved training stage 1, where the crows had to take one of two tools positioned together to an out-of-sight apparatus, they took much longer in training stage 2, where they had to take tools positioned inside different compartments. On average, the crows made 8 errors at stage 1 before reaching criterion but 30.7 errors at stage 2. Thus, despite both these tasks requiring mental representation, choosing which compartment to take a tool from, rather than choosing between two tools in the same compartment, appears to have been much more cognitively demanding, possibly due to the increased working memory load this task required. Without training stage 2, it seems likely that the crows would have failed the experiment 1, due to the extra demand imposed by having to take one of two tools positioned inside different compartments of the shield to an apparatus. Once crows had learned to do this, however, they were able to mentally represent the location and identity of tools and apparatuses while planning and performing a three-stage behavioral sequence involving metatool use.

Interestingly, the crows did not perform as well on any of the stone metatool tasks we presented to them. There are a number of possible explanations for this. One is that it was harder for the crows to be performing stone- rather than stick-tool use while making a decision. Tool use itself has been shown to be cognitively demanding for animals [52], so it is possible that some forms of tool use may in themselves be more demanding than others. For example, holding the stone may have required more attention or motor control, either due to its affordances or because the crows had less practice holding stones than sticks due to using sticks, but not stones, in the wild. A second possibility is that stones were not as easy to represent as the starting decision point in a sequence compared to sticks, which had a downstream effect on the crows' ability to preplan a sequence. Still, the crows' performance in solving the stick metatool problem, where they had to mentally represent the location of a stone tool, clearly shows that their ability to plan is flexible enough to include tools they have been trained to use, such as stones, rather than those they have evolved to use, such as sticks.

Our results support the hypothesis that NC crows can use mental trial and error when solving metatool problems and so can act as Popperian creatures. The birds represented different states of the world (the location and identity of the functional and distractor tools, the overall goal, and the location and identity of the different apparatus) and then used these representations to plan out a sequence of behaviors toward an overall goal before finally executing this plan. However, the nature of the representations that the crows are using is unclear. Are these representations of the crows' entire trajectory or just key decision points? Are they based only on semantic knowledge of likely outcomes, or do they incorporate episodic elements as well? Such questions will be a focus of future work.

STAR★METHODS

Detailed methods are provided in the online version of this paper and include the following:

- KEY SOURCE TABLE
- CONTACT FOR REAGENT AND RESOURCE SHARING
- EXPERIMENTAL MODEL AND SUBJECT DETAILS

- METHOD DETAILS
  - Procedure
  - Prior Experience
- QUANTIFICATION AND STATISTICAL ANALYSIS

SUPPLEMENTAL INFORMATION

Supplemental Information includes one figure, four tables, one video, and one data file and can be found with this article online at https://doi.org/10.1016/j.cub.2019.01.008.

ACKNOWLEDGMENTS

We thank Province Sud for granting us permission to work in New Caledonia and Dean M. and Boris C. for allowing us access to their properties for catching and releasing the crows. Many thanks to Rachel Johnston for help with video coding and Tom Vardy for statistical advice and three anonymous reviewers for their helpful comments on this paper. This research was supported by a Royal Society of New Zealand Rutherford Discovery Fellowship and a Prime Minister's McDiarmid Emerging Scientist Prize (A.H.T.), the European Research Council under the European Union's Seventh Framework Programme (FP7/2007-2013)/ERC Grant Agreement No. 3399933 (N.S.C.), and the Max Planck Institute for the Science of Human History (R.D.G.).

AUTHORS CONTRIBUTION

Conceptualization, R.G., M.S., M.B., R.D.G., N.S.C., and A.H.T.; Methodology, R.G., M.S., M.B., and A.H.T.; Investigation, R.G., M.S., M.B., and A.F.; Formal Analysis, R.G.; Writing – Original Draft, R.G. and A.H.T.; Writing – Review & Editing, R.G., M.S., M.B., A.F., R.M., R.D.G., N.S.C., and A.H.T.; Supervision, A.H.T.; Funding Acquisition, R.D.G., N.S.C., and A.H.T.; Resources, R.D.G., N.S.C., and A.H.T.

DECLARATION OF INTERESTS

The authors declare no competing interests.

Received: August 6, 2018
Revised: November 22, 2018
Accepted: January 3, 2019
Published: February 7, 2019

REFERENCES

1. Ward, G., and Allport, A. (1997). Planning and Problem solving Using the Five disc Tower of London Task. Q. J. Exp. Psychol. Sect. A *50*, 49–78.
2. Hodgson, T.L., Bajwa, A., Owen, A.M., and Kennard, C. (2000). The strategic control of gaze direction in the Tower-of-London task. J. Cogn. Neurosci. *12*, 894–907.
3. Unterrainer, J.M., Rahm, B., Leonhart, R., Ruff, C.C., and Halsband, U. (2003). The Tower of London: the impact of instructions, cueing, and learning on planning abilities. Brain Res. Cogn. Brain Res. *17*, 675–683.
4. Redshaw, J., Taylor, A.H., and Suddendorf, T. (2017). Flexible Planning in Ravens? Trends Cogn. Sci. *21*, 821–822.
5. Scarf, D., and Colombo, M. (2009). Eye movements during list execution reveal no planning in monkeys (*Macaca fascicularis*). J. Exp. Psychol. Anim. Behav. Process. *35*, 587–592.
6. Suddendorf, T., and Corballis, M.C. (2007). The evolution of foresight: What is mental time travel, and is it unique to humans? Behav. Brain Sci. *30*, 299–313, discussion 313–351.
7. Taylor, A.H., Elliffe, D., Hunt, G.R., and Gray, R.D. (2010). Complex cognition and behavioural innovation in New Caledonian crows. Proc. Biol. Sci. *277*, 2637–2643.
8. Taylor, A.H., Hunt, G.R., Holzhaider, J.C., and Gray, R.D. (2007). Spontaneous metatool use by New Caledonian crows. Curr. Biol. *17*, 1504–1507.
9. Völter, C.J., and Call, J. (2014). Younger apes and human children plan their moves in a maze task. Cognition *130*, 186–203.
10. Camp, E., and Shupe, E. (2017). Instrumental reasoning in nonhuman animals. In The Routledge Handbook of Philosophy of Animal Minds, K. Andrews, and J. Beck, eds. (Routledge), pp. 100–108.
11. Pfeiffer, B.E., and Foster, D.J. (2013). Hippocampal place-cell sequences depict future paths to remembered goals. Nature *497*, 74–79.
12. Dragoi, G., and Tonegawa, S. (2013). Distinct preplay of multiple novel spatial experiences in the rat. Proc. Natl. Acad. Sci. USA *110*, 9100–9105.
13. Ólafsdóttir, H.F., Barry, C., Saleem, A.B., Hassabis, D., and Spiers, H.J. (2015). Hippocampal place cells construct reward related sequences through unexplored space. eLife *4*, e06063.
14. Silva, D., Feng, T., and Foster, D.J. (2015). Trajectory events across hippocampal place cells require previous experience. Nat. Neurosci. *18*, 1772–1779.
15. Bendor, D., and Spiers, H.J. (2016). Does the Hippocampus Map Out the Future? Trends Cogn. Sci. *20*, 167–169.
16. Eichenbaum, H. (2015). Does the hippocampus preplay memories? Nat. Neurosci. *18*, 1701–1702.
17. Janson, C. (2014). Death of the (traveling) salesman: primates do not show clear evidence of multi-step route planning. Am. J. Primatol. *76*, 410–420.
18. Kabadayi, C., and Osvath, M. (2017). Ravens parallel great apes in flexible planning for tool-use and bartering. Science *357*, 202–204.
19. Martin-Ordas, G., Schumacher, L., and Call, J. (2012). Sequential tool use in great apes. PLoS ONE *7*, e52074.
20. Millikan, R.G. (2006). Styles of Rationality. In Rational Animals?, M. Nudds, and S. Hurley, eds. (Oxford University Press).
21. Mulcahy, N.J., and Call, J. (2006). Apes Save Tools for Future Use. Science *312*, 1038–1040.
22. Raby, C.R., Alexis, D.M., Dickinson, A., and Clayton, N.S. (2007). Planning for the future by western scrub-jays. Nature *445*, 919–921.
23. Bird, C.D., and Emery, N.J. (2009). Insightful problem solving and creative tool modification by captive nontool-using rooks. Proc. Natl. Acad. Sci. USA *106*, 10370–10375.
24. Scarf, D., and Colombo, M. (2010). Representation of serial order in pigeons (Columba livia). J. Exp. Psychol. Anim. Behav. Process. *36*, 423–429.
25. Köhler, W. (1925). The Mentality of Apes (London: Routledge & Kegan Paul).
26. Bird, C.D., and Emery, N.J. (2009). Rooks use stones to raise the water level to reach a floating worm. Curr. Biol. *19*, 1410–1414.
27. Hanus, D., Mendes, N., Tennie, C., and Call, J. (2011). Comparing the performances of apes (*Gorilla gorilla, Pan troglodytes, Pongo pygmaeus*) and human children (*Homo sapiens*) in the floating peanut task. PLoS ONE *6*, e19555.
28. Jelbert, S.A., Taylor, A.H., Cheke, L.G., Clayton, N.S., and Gray, R.D. (2014). Using the Aesop's fable paradigm to investigate causal understanding of water displacement by New Caledonian crows. PLoS ONE *9*, e92895.
29. Auersperg, A.M.I., Kacelnik, A., and von Bayern, A.M.P. (2013). Explorative learning and functional inferences on a five-step means-means-end problem in Goffin's cockatoos (*Cacatuagoffini*). PLoS ONE *8*, e68979.
30. Sabbatini, G., Manrique, H.M., Trapanese, C., De Bortoli Vizioli, A., Call, J., and Visalberghi, E. (2013). Sequential use of rigid and pliable tools in tufted capuchin monkeys (Sapajus spp.). Anim. Behav. *87*, 213–220.
31. Weir, A.A.S., Chappell, J., and Kacelnik, A. (2002). Shaping of hooks in New Caledonian crows. Science *297*, 981.
32. Seed, A.M., Tebbich, S., Emery, N.J., and Clayton, N.S. (2006). Investigating physical cognition in rooks, Corvus frugilegus. Curr. Biol. *16*, 697–701.
33. Mulcahy, N.J., and Call, J. (2006). How great apes perform on a modified trap-tube task. Anim. Cogn. *9*, 193–199.

CellPress

34. Taylor, A.H., Hunt, G.R., Medina, F.S., and Gray, R.D. (2009). Do new caledonian crows solve physical problems through causal reasoning? Proc. Biol. Sci. *276*, 247–254.

35. Visalberghi, E., and Limongelli, L. (1994). Lack of comprehension of cause-effect relations in tool-using capuchin monkeys (*Cebus apella*). J. Comp. Psychol. *108*, 15–22.

36. Beran, M.J., Parrish, A.E., Futch, S.E., Evans, T.A., and Perdue, B.M. (2015). Looking ahead? Computerized maze task performance by chimpanzees (*Pan troglodytes*), rhesus monkeys (*Macaca mulatta*), capuchin monkeys (*Cebus apella*), and human children (*Homo sapiens*). J. Comp. Psychol. *129*, 160–173.

37. Tecwyn, E.C., Thorpe, S.K.S., and Chappell, J. (2013). A novel test of planning ability: great apes can plan step-by-step but not in advance of action. Behav. Processes *100*, 174–184.

38. Dennett, D.C. (1995). Darwin's Dangerous Idea: Evolution and the Meanings of Life (Simon & Schuster).

39. Taylor, A.H., Hunt, G.R., and Gray, R.D. (2012). Context-dependent tool use in New Caledonian crows. Biol. Lett. *8*, 205–207.

40. Taylor, A.H., and Gray, R.D. (2009). Animal cognition: Aesop's fable flies from fiction to fact. Curr. Biol. *19*, R731–R732.

41. Balleine, B.W., Garner, C., Gonzalez, F., and Dickinson, A. (1995). Motivational Control of Heterogeneous Instrumental Chains. J. Exp. Psychol. Anim. Behav. Process. *21*, 203–217.

42. Shettleworth, S.J. (2012). Do animals have insight, and what is insight anyway? Can. J. Exp. Psychol. *66*, 217–226.

43. Reed, P., and Morgan, T.A. (2007). Resurgence of behavior during extinction depends on previous rate of response. Learn. Behav. *35*, 106–114.

44. Thorndike, E.L. (1911). Animal Intelligence (Darien, CT: Hafner Publishing).

45. Biro, D., and Matsuzawa, T. (1999). Numerical Ordering in a Chimpanzee (*Pan troglodytes*): Planning, Execution, and Monitoring. J. Comp. Psychol. *113*, 178–185.

46. Hunt, G.R. (1996). Manufacture and use of hook-tools by New Caledonian crows. Nature *379*, 249–251.

47. Wimpenny, J.H., Weir, A.A.S., Clayton, L., Rutz, C., and Kacelnik, A. (2009). Cognitive processes associated with sequential tool use in New Caledonian crows. PLoS ONE *4*, e6471.

48. Clayton, N. (2007). Animal cognition: crows spontaneously solve a meta-tool task. Curr. Biol. *17*, R894–R895.

49. Taylor, A.H., Medina, F.S., Holzhaider, J.C., Hearne, L.J., Hunt, G.R., and Gray, R.D. (2010). An investigation into the cognition behind spontaneous string pulling in New Caledonian crows. PLoS ONE *5*, e9345.

50. Habl, C., and Auersperg, A.M.I. (2017). The keybox: Shape-frame fitting during tool use in Goffin's cockatoos (*Cacatua goffiniana*). PLoS ONE *12*, e0186859.

51. Laumer, I.B., Bugnyar, T., and Auersperg, A.M.I. (2016). Flexible decision-making relative to reward quality and tool functionality in Goffin cockatoos (*Cacatua goffiniana*). Sci. Rep. *6*, 28380.

52. Seed, A.M., Call, J., Emery, N.J., and Clayton, N.S. (2009). Chimpanzees solve the trap problem when the confound of tool-use is removed. J. Exp. Psychol. Anim. Behav. Process. *35*, 23–34.

53. Kenward, B., Rutz, C., Weir, A.A.S., Chappell, J., and Kacelnik, A. (2004). Morphology and sexual dimorphism of the New Caledonian Crow *Corvus moneduloides*, with notes on its behaviour and ecology. Ibis *146*, 652–660.

54. R Development Core Team (2016). R: A Language and Environment for Statistical Computing (R Foundation for Statistical Computing).

55. Gamer, M., Lemon, J., and Singh, P. (2012). irr: Various Coefficients of Interrater Reliability and Agreement. Available at http://www.r-project.org.

STAR★METHODS

KEY SOURCE TABLE

REAGENT or RESOURCE	SOURCE	IDENTIFIER
Experimental Models: Organisms/Strains		
Corvus moneduloides	Wild-caught crows, Moindou, Grand Terre, New Caledonia	N/A

CONTACT FOR REAGENT AND RESOURCE SHARING

Further information and requests for resources and reagents should be directed to and will be fulfilled by the Lead Contact, Romana Gruber (rgru908@aucklanduni.ac.nz).

EXPERIMENTAL MODEL AND SUBJECT DETAILS

The study was carried out with fourteen wild caught New Caledonian crows (*Corvus moneduloides*) on the island of Grand Terre, New Caledonia. Eight of the crows were adults more than 2 years old (Mercury, Io, Saturn, Uranus, Janis, David, Elvis, and Bob), and six were juveniles less than one year old (Neptune, Triton, Mars, Venus, Freddie & Aretha). Based on sexual size dimorphism [53], six crows were identified as females (Mercury, Neptune, Triton, Uranus, Janis and Aretha), and the other eight as males. The crows were kept in family groups and housed for five months in a 10 cage outdoor aviary, with each cage measuring at least 2 × 3 x 3 m. They had access to water *ad libitum,* and were fed fruits and dog biscuits soaked in water. Small pieces of meat were used as rewards. All testing happened in a compartment with no visual access to the other crows. Our work was carried out under the approval of the University of Auckland Animal Ethics Committee (reference no. 001823).

METHOD DETAILS

We used three types of apparatus: a wooden screen, stone dropping boxes and horizontal Perspex tubes. The wooden screen (50 × 50 × 40 cm) functioned as a visual shield and consisted of three large compartments (25 × 25 × 40 cm) on three sides of the box, and one small compartment (5 × 5 x 10 cm) on the fourth side of the box, which was the starting position for the crows (Figure 2). The stone dropping boxes (henceforth called stone apparatus) were similar to those used in previous studies [23, 28] and measured 16 × 10 × 10 cm. They were made out of clear Perspex, with the collapsing trap-platform in the box colored in white for better visibility of the reward and the tool. On top of the box was a 12 cm long tube with a diameter of 5 cm and a slant of 30° in the middle, to prevent stick tools being pushed down the tube contacting the platform. The crows were therefore only able to release the platform by dropping a stone down the tube. The horizontal Perspex tubes (henceforth called the stick apparatus) were 18 cm long with a diameter of 5 cm, mounted 8 cm above a base. One of these tubes had two 1 × 3 cm holes drilled into them close to the center, which made it easier for crows to lever out heavy stones positioned in the center of the tube. The second tube had a continuous surface and contained a stick tool or the food reward, depending on the task it was presented in.

Procedure

Training

All crows received two training steps in this study. Training Stage 1 examined if the crows could mentally represent the identity of the goal (the stone and stick apparatus) while moving around the wooden shield. To do this we placed a stick tool and a stone tool in one large compartment and either the stone or stick apparatus in another large compartment. The apparatus and both tools in the large compartments were randomized between trials. Due to the structure of the wooden shield the crows could not simultaneously see the contents of more than one compartment. To solve the task the birds had to choose the correct tool and take it to the apparatus. If they chose the wrong tool and used it to make contact with the apparatus this was counted as an error. In 2017, subjects were also allowed to pick up one of the two tools, take it to the apparatus, return with this tool and swap it for the second before returning the apparatus, as this tool swap required them to keep in mind the apparatus type. They were not allowed to take a (wrong) tool to the apparatus, drop it and then return to take the second tool, as the crow would be able to see the apparatus and both tools at the same time if they did this. Therefore, the trial was interrupted by the experimenter knocking on the door and going into the compartment and counted as an error. In 2018 however, if subjects chose the wrong tool, took it to the apparatus, but then did not interact and tried to return to get the second tool, the trial was interrupted and this was counted as an error. This gave the crows experience that their first choice had to be correct in order to get rewarded. In 2017, crows were given blocks of 12 trials and had to reach a criterion of 18 out of 24 correct trials in two consecutive blocks (binomial test with alpha set at 0.05) in order to move on to Training Stage 2. This was changed to blocks of 10 trials with a criterion of 16/20 in 2018. Training Stage 2 was the same as Training 1, in that crows had to

mentally represent the apparatus while moving around the shield, with the difference that both tools were located in different compartments (position of apparatus and both tools was randomized and counterbalanced across trials). The same rules as in Training 1 applied for Training 2, so 2017 birds were allowed to take the tool to the apparatus and then return to swap it if they did not interact with the apparatus, while 2018 birds were not. The training was completed when the 2017 crows reached the criterion of 18 out of 24 correct trials in two consecutive blocks of 12 trials (binomial test with alpha set at 0.05). This was changed to blocks of 10 trials with a criterion of 16/20 in 2018.

Experiments

In Experiment 1 the crows received two conditions where they had to mentally represent and then solve two different 3-stage metatool problems. In the first problem, crows had to use a stick to gain access to a stone that then could be used to gain food (Figure 2A). In the second problem, crows had to use a stone to gain access to a stick that could then be used to gain food (Figure 2B). Each stage of the problem was housed in one of the three large compartments of the wooden shield (Figure 1), meaning that the crows were unable to see more than one stage of the problem simultaneously. At the start of a trial the crows entered the testing room and observed that the small compartment of the wooden shield, which would later house a tool, was empty. They were then allowed to inspect the rest of the experimental setup for 1 min. If they did not inspect each large compartment, each side of the wooden screen was baited with a small piece of meat to ensure crows had observed each problem stage. After the inspection, the experimenter entered the room, and placed the starting tool in the small compartment. The crow could now pick up this tool and use it to extract the tools housed in the apparatus. The time between inspecting the set up and picking up the first tool was not more than 15 s. Crows were given trials of the first condition until they reached a criterion of 16 out of 20 correct trials (binomial test with alpha set at 0.015) or until they had been presented with 40 trials. If crows scored 8/10 or in their final block of the 40 trials, we continued testing for a final block to see if they would reach criterion.

Crows were then given trials of the second condition. In 2017 conditions were randomized between crows while in 2018 crows received the stick condition then the stone condition. We made this change because of how difficult the 2017 crows had found the stone condition to solve. We hoped presenting the experiments in this order would increase the chance of crows' successfully solving the stone problem and so allow us to run this study quicker, creating time for other experiments (such as Experiment 2 and 3) to be run before the crows were released back into the wild. Within both conditions the position of the functional and distractor apparatuses were counterbalanced and randomized between trials. The location of the initial tool and food was always in the same position across trials. A trial was counted as a failure and was interrupted by the experimenter if the crow (a) took the initial tool to the distractor apparatus containing the same tool type (a first action error), (b) obtained the functional tool with the initial tool but then inserted it in the distractor apparatus (a second action error), or (c) took the initial tool directly to the final apparatus containing food (a third action error). In 2017, it was not counted as an error if the crow took a tool to an apparatus but did not interact with it, and then took the tool to the other apparatus. In 2018, this was counted as an error, with the trial being stopped if the crow did attempt this behavior (Video S1, Experiment 1).

Experiment 2 was highly similar to Experiment 1 except that within any one block crows were given three trials types: a stick metatool trial identical to the set-up presented in Experiment 1, a stone metatool trial identical to the set-up presented in Experiment 1 and a novel shortcut trial. In this latter trial each crow had to take a stone directly to the baited platform apparatus while ignoring two sticks placed inside the two sub-goal apparatus (tube and platform), as these sticks were non-functional for the overall goal. Crows therefore had to represent both the sub-goal and goal of the problem in order to solve these trials. Crows were given blocks of trials until they reached a criterion of 16 out of 20 correct trials (binomial test with alpha set at 0.015) or until they had been presented with 120 trials. Note it was not possible to run a stick short cut condition where crows would have needed to take a short stick tool directly to a tube apparatus, while ignoring two apparatus containing stones. This is because we would have had to change the stick size: the short stick would have been non-functional for the tube apparatus baited with food, as it was too short. Changing the stick size would have made it impossible to know if successful performances were because crows were representing the final goal, or simply changing their behavior because the stick size had changed (Video S1, Experiment 2).

Experiment 3 was a repeat of Experiment 1 with one key difference: instead of two different tools being presented in the same sub-goal apparatus, now the same tool was presented in the two different sub-goal apparatuses. Two crows (Mercury, Triton) were first given trials of the stick condition (where they had to use a stick to get a stone from a tube while avoiding a stone in a platform apparatus), until they reached a criterion of 16 out of 20 correct trials (binomial test with alpha set at 0.015). They were then tested with the stone condition, where they had to take a stone to get a stick from platform apparatus, while avoiding a stone in a tube. Again, they were tested until they reached the same criterion. One crow, Mars, was given the stone condition first, and then the stick condition (Video S1, Experiment 3).

Prior Experience

None of the crows had prior experience of mentally representing multi-stage tool problems, or of using tools as metatools, before being given Experiments 1-3. However, crows were given a number of different tasks involving tool use before being participating in this study. Five of the six 2017 crows (Aretha being the exception) participated in a tool functionality study similar to [51] where they learnt to choose the correct tool for the right job when presented with the stone apparatus, the stick apparatus and both stick and stone tools (see SI for further details, see figure S1). The 2018 crows received the same tool functionality problems, aside from the motivation and tool functionality conditions, due to time constraints. Additionally, there was one key change to the criterion used throughout these 2018 tests: if the crows chose a tool, then took it to an apparatus but did not interact with it, and then attempted

to swap tools, this was counted as an error, unlike in 2017. All 2017 and 2018 crows were also presented with two problems where the stick and stone tools were presented out-of-sight of the apparatus, using a wooden screen (see SI for further details and diagram). These problems where highly similar to the training stages we describe above, with the key difference being that barriers were used, rather than the shield we used throughout our experiment. Three 2017 (Janice, David and Freddie) and two 2018 crows (Mercury and Uranus) had also been given problems where they had to choose a tool, and after a time delay, take the tool to an apparatus to gain food. In Condition 1, crows were shown a stick apparatus (described above) that was baited with food in Compartment 1.The crows were then moved to Compartment 2. Here, the crows were presented with various objects, including a stick tool. After the crows had chosen one of these objects they were given access to Compartment 1 again. Choosing the stick tool in Compartment 2, rather than the other objects, and then transporting it back to Compartment 1, allowed the crows to access the reward. During testing, crows were presented with novel-tool apparatus combinations but the same temporal pattern of events.

QUANTIFICATION AND STATISTICAL ANALYSIS

Trials were video recorded from four different angles (two Sony 4K XAVC S, one Panasonic HC-X920M, and one GoPro Hero 3+). All statistical tests were conducted in R [54] and are two-tailed. Twenty percent of the videos from all four angles were coded by a blind coder and the inter-observer reliability was 1 (Cohen's Kappa, R package 'irr' [55]). Binomial analyses were run, with the probability of a correct choice at 0.5.

Current Biology, Volume *29*

Supplemental Information

New Caledonian Crows Use Mental Representations to Solve Metatool Problems

Romana Gruber, Martina Schiestl, Markus Boeckle, Anna Frohnwieser, Rachael Miller, Russell D. Gray, Nicola S. Clayton, and Alex H. Taylor

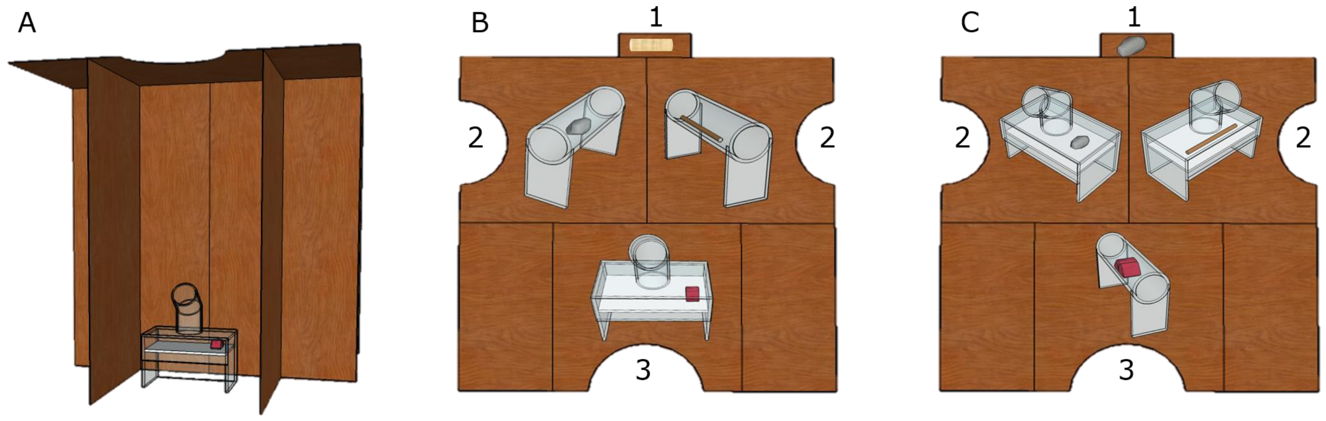

Figure S1. Diagram of the wooden box used to visually shield off the apparatuses from each other (related to Figure 1). Viewed from the side (A), and from a bird's eye view (B & C). 1) Initial tool starting position, 2) Sub-goals 3) Goal. Red block indicates meat position.

		Number of Blocks for Training Stage 1					
**Year**	**Individuals**	**1**	**2**	**3**	**4**	**5**	**6**
2017	**Janis**	9/12	9/12				
2017	**David**	8/12	10/12				
2017	**Freddie**	11/12	10/12				
2017	**Elvis**	10/12	8/12				
2017	**Bob**	10/12	11/12				
2017	**Aretha**	7/12	6/12	6/12	5/12	8/12	10/12
2018	**Mercury**	6/10	8/10	6/10	8/10	9/10	
2018	**Io**	7/10	10/10				
2018	**Neptune**	7/10	10/10				
2018	**Triton**	8/10	6/10	9/10	6/10	7/10	10/10
2018	**Mars**	7/10	9/10				
2018	**Saturn**	10/10	9/10				
2018	**Uranus**	7/10	8/10	8/10			
2018	**Venus**	7/10	6/10	8/10	7/10	9/10	

Table S1. Blocks required for 2017 and 2018 birds for Training Stage 1 (related to Figure 2). Note birds were given blocks of 12 trials in 2017, and needed to reach a criterion of 18/24, while in 2018 they were given blocks of 10 trials, and needed to reach a criterion of 16/20. The colours indicate a failed block (red) and a successfully solved block (green).

		Number of Blocks for Training Stage 2																	
**Year**	**Individuals**	**1**	**2**	**3**	**4**	**5**	**6**	**7**	**8**	**9**	**10**	**11**	**12**	**13**	**14**	**15**	**16**	**17**	**18**
2017	**Janis**	5/12	9/12	7/12	11/12														
2017	**David***	7/12	10/12																
2017	**Freddie**	7/12	9/12	5/12	5/12	7/12	10/12	8/12											
2017	**Elvis**	7/12	9/12	5/12	7/12	10/12	7/12	9/12	8/12	10/12									
2017	**Bob**	6/12	3/12	5/12	5/12	7/12	6/12	6/12	6/12	7/12	8/12	10/12							
2017	**Aretha**	6/12	5/12	5/12	8/12	4/12	6/12	11/12	9/12										
2018	**Mercury**	6/10	7/10	5/10	4/10	7/10	7/10	6/10	6/10	8/10	8/10	7/10	8/10	10/10	8/10	9/10			
2018	**Io**	10/10	7/10	6/10	7/10	8/10	7/10	7/10	5/10	10/10	7/10								
2018	**Neptune**	8/10	8/10	4/10	6/10	8/10	9/10												
2018	**Triton**	6/10	8/10	5/10	1/10	4/10	8/10	7/10	4/10	5/10	5/10	9/10	7/10	7/10	9/10	10/10			
2018	**Mars**	9/10	10/10	9/10	8/10														
2018	**Saturn**	6/10	8/10	10/10	10/10	9/10													
2018	**Uranus**	7/10	7/10	10/10	3/10	6/10	10/10												
2018	**Venus**	5/10	5/10	6/10	4/10	6/10	7/10	5/10	7/10	8/10	5/10	7/10	7/10	8/10	6/10	8/10	6/10	8/10	9/10

*David reached criterion in 17 out of 24 correct trials due to time constraints.

Table S2. Blocks required for 2017 and 2018 birds for Training Stage 2 (related to Figure 2). Note birds were given blocks of 12 trials in 2017, and needed to reach a criterion of 18/24, while in 2018 they were given blocks of 10 trials, and needed to reach a criterion of 16/20. The colours indicate a failed block (red) and a successfully solved block (green).

	Training	Experiment 1: Sub-goal Representation			Experiment 2: Goal Representation	Experiment 3: Apparatus Representation		
**Choices**		**Stick**	**Stone**	**Combined**		**Stick**	**Stone**	**Combined**
**Correct**	69%	66.26%	48.08%	55.7%	61.55%	85.32%	59.83%	72.58%
**1st action error**	NA	27.91%	46.68%	38.82%	30.18%	13.17%	37.38%	25.26%
**2nd action error**	NA	1.94%	1.75%	1.83%	0%	1.59%	0.56%	1.07%
**3rd action error**	NA	3.88%	3.50%	3.66%	8.27%	0%	2.23%	1.12%
**Adults–correct**	73.81%	69.85%	40.42%	51.84%	58.84%	90%	56.67%	65%
**Juveniles-correct**	67.52%	69.27%	58.14%	61.39%	66.39%	82.50%	61.02%	66.45%

Table S3. Percentages of correct choices and errors within Training and the three experiments (related to Figures 2, 3 and 4).

Individuals	Stick Condition	Stone Condition
Aretha	30%	37.8%
Freddie	14.3%	58.1%
Elvis	48.8%	35%
Janis	40.9%	35%
David	50%	2.5%
Bob	50%	57.5%

Table S4. Inspection frequency of the apparatuses with tool in beak by the 2017 crows, for both conditions (related to Figure 2).
